# Eating Right, Sleeping Tight? A Cross-Sectional Study on the Student-Athlete Paradox for Diet and Sleep Behaviors

**DOI:** 10.3390/nu17182946

**Published:** 2025-09-12

**Authors:** Olga Papale, Emanuel Festino, Francesca Di Rocco, Marianna De Maio, Carl Foster, Cristina Cortis, Andrea Fusco

**Affiliations:** 1 Department of Human Sciences, Society and Health, University of Cassino and Lazio Meridionale, 03043 Cassino, Italy; olga.papale@unicas.it (O.P.); emanuel.festino@unicas.it (E.F.); francesca.dirocco@uniroma5.it (F.D.R.); marianna.demaio@unicas.it (M.D.M.); 2 European University of Technology EUt+, 03043 Cassino, Italy; 3 Department of Human Sciences and Promotion of the Quality of Life, “San Raffaele” Open University of Rome, 00166 Rome, Italy; 4 Department of Exercise and Sport Science, University of Wisconsin-La Crosse, La Crosse, WI 54601, USA; cfosteruwl@gmail.com; 5 Department of Medicine and Aging Sciences, University “G. d’Annunzio” of Chieti-Pescara, 66100 Chieti, Italy; andrea.fusco@unich.it

**Keywords:** Mediterranean diet, physical activity, sedentary behaviors, sleep quality, collegiate athletes

## Abstract

**Background:** Student-athletes face the dual challenge of balancing academic and athletic commitments, which may simultaneously promote healthy lifestyle habits while increasing psychosocial and physiological stressors, particularly among female student-athletes. Understanding how these competing demands affect key behavioral (e.g., dietary habits, sleep, and chronotype) and psychological (e.g., body image) factors is essential for supporting their overall well-being. Therefore, this cross-sectional study investigated body dissatisfaction, adherence to the Mediterranean diet, sleep quality, and chronotype in female student-athletes compared to sedentary peers. **Methods:** Twenty-eight female participants voluntarily participated in the study. Twelve volleyball student-athletes (age 21.6 ± 2.4 years) were assessed during their competitive in-season period, and sixteen non-athlete students with a high sitting time (age 24.0 ± 3.2 years) completed the Mediterranean Diet Adherence questionnaire (PREDIMED), Pittsburgh Sleep Quality Index (PSQI), Morningness–Eveningness Questionnaire (MEQ), and Body Image Dimensional Assessment (body dissatisfaction) to assess their overall well-being. **Results:** Student-athletes showed significantly (*p* < 0.05) higher adherence to the Mediterranean diet (PREDIMED: 8.5 ± 1.5 score), although experiencing poorer sleep quality (PSQI: 6.8 ± 3.0 score) compared to non-athlete students with higher sitting times (PREDIMED: 6.7 ± 1.6 score; PSQI: 4.6 ± 2.3 score). Conversely, comparative body dissatisfaction was significantly higher in non-athlete students with a high sitting time (19.4 ± 24.5%) than in student-athletes (5.6 ± 10.5%). No significant differences emerged for chronotype or overall body dissatisfaction. **Conclusions**: These findings highlight a paradoxical health pattern in female student-athletes who combine healthier eating habits with poorer sleep quality. The results emphasize the importance of comprehensive wellness strategies that integrate dietary habits, sleep hygiene, and psychophysiological factors to better support female student-athletes in managing dual-career demands.

## 1. Introduction

Student-athletes represent a specific population facing unique physical, psychological, and social demands. To achieve holistic development, student-athletes have the right to combine their higher sport and education careers (e.g., dual career), both of which are relevant to empowering their future role in society, especially at the end of their competitive sport period [1]. Interest in this population has increased in recent years, driven in part by the growing attention from organizations such as the European Athlete as Student (EAS) Network [2] and the European Commission [3]. Moreover, scholars introduced the concept of dual-career balance, defined as “a combination of sport and studies that helps them to achieve their educational and athletic goals, live satisfying private lives, and maintain their health and well-being” [4].

Originally, sport participation, and specifically the presence of student-athletes, was predominantly male-oriented. However, in recent years, female participation in high-level sport (e.g., Olympic level) has progressively increased, approaching parity with male counterparts [5], with a significant number of student-athletes [6]. However, female representation remains unequal in sports research [7], which tend to focus on male performance rather than the general well-being of student-athletes. In this context, well-being should be understood not only in terms of physical health but also in psychological resources that sustain athletes across domains. Accordingly, in the last decade, mental health has emerged as a key resource for achieving both academic and athletic success [8].

The World Health Organization (WHO) defines mental health as a state of well-being that enables people to cope with life stresses, achieve their goals, work productively and contribute to their community [1]. This concept is closely linked to quality of life, reflecting an individual’s perception of their position in life in the context of their goals, expectations, standards, and concerns [9]. Regular participation in physical activity, structured exercise, and sports, typical for student-athletes, has been shown to reduce anxiety and depression, enhance confidence, and improve mood [10], particularly in females [11]. In contrast, prolonged sitting time and sedentary behaviors can negatively affect mental health and quality of life [12].

Optimal mental health and athletic performance cannot be sustained by focusing on a single behavior. Instead, a combination of interconnected lifestyle factors, including physical activity and exercise, sleep, dietary habits, and body image, collectively influences psychological well-being, physical recovery, cognitive functioning, and overall quality of life in student-athletes. Disruptions in one area often affect others, warranting multidimensional health promotion strategies [13]. Student-athletes generally adopt healthy habits such as adherence to a balanced diet like the Mediterranean diet, which is known to support physical recovery, mental health, and positive body image. However, sleep quality is often poor in this population [14] and age group [15], creating a paradox where healthy nutrition contrasts with inadequate rest, potentially limiting the benefits of both.

Previous study [16] has shown that sleep deprivation and chronic sleep loss negatively affect cognitive performance, learning, memory, reaction time, vigilance, mood, recovery, and physical performance, particularly in strength-based activities, while also being associated with metabolic and endocrine alterations. Optimal sleep is thus vital for athletes since sports performance depends on cognitive, physiological, and physical integration. Maximizing athletic performance is a primary objective for many student-athletes, particularly those aiming for athletic scholarships or professional careers. Sleep deprivation negatively affects anaerobic capacity, sport-specific skills, and neurocognitive functioning [17], highlighting the essential role of adequate sleep quality and duration in training, recovery, mood regulation, and overall sports performance [18].

Dual-career athletes, when compared to their student peers, might be more likely to present with sleep problems since they manage additional social roles and significant time demands, potentially negatively impacting sleep patterns and recovery processes [19]. Moreover, academic and training schedules may not align with their natural sleep preferences or chronotype (e.g., an individual’s preferred timing for sleep and daily activities within a 24 h cycle), which might influence sleep quality, sleep duration, and social jet lag, particularly among populations with irregular schedules. Furthermore, it affects daytime functioning, including academic performance, daytime sleepiness, and work-related fatigue, and has been associated with various physical and mental health aspects, such as metabolism and dietary habits [20]. Optimum nutrient intake and good dietary habits have been recognized as key factors in improving athletic performance in terms of quality of training and speedy recovery from exercise in athletes. Variable results have been reported regarding nutrition knowledge of student-athletes, with good dietary knowledge and habits positively associated with better general mental health outcomes [21], as well as a more positive body perception and body image [22], improved mood, and reduced perceived stress. These effects might be especially relevant for athletes who are exposed to both physical–psychological demands and academic responsibilities. A balanced and nutrient-rich diet, such as the Mediterranean diet, may support enhanced recovery, reduced inflammation, and improved overall performance [23]. Moreover, evidence suggests that recreational physical activity mediates the association between dietary intake of live microbes and the systemic immune-inflammation index, indicating that the favorable link between diet quality and inflammatory burden may be partly realized through physical activity, thereby supporting an integrated lifestyle framework for understanding student-athletes’ health behavior patterns [24,25]. Moreover, a close connection emerged between dietary habits, and body image [26], which is a multidimensional construct comprising a behavioral aspect related to body-related behaviors (e.g., checking behaviors), a perceptual aspect regarding body characteristic perceptions (e.g., estimating one’s body size or weight), and a cognitive–affective aspect involving thoughts and feelings toward one’s body. Body image, dietary adherence, sleep quality, and chronotype represent critical factors that may influence both the physical performance and psychological well-being of athletes, especially student-athletes, further highlighting the need for a multidimensional approach. Although these aspects play a key role in promoting health, there is still a lack of research simultaneously addressing these interconnected dimensions, especially in dual-career athletes. Thus, it remains unclear whether these dimensions are considered in promoting the health and performance of student-athletes, which is particularly concerning given recent evidence of persistent gender disparities in sport and exercise science research [27]. This disparity in research representation raises critical questions about the understanding of female student-athletes’ experiences and needs, particularly in the field of mental health and well-being. This limits our understanding of how these interconnected lifestyle dimensions influence well-being and performance in dual-career female athletes, which is a group facing unique physical, psychological, and social demands.

To address this gap, the present study aimed to evaluate adherence to the Mediterranean diet, sleep quality, chronotype and body dissatisfaction among female student-athletes compared to sedentary peers. We hypothesized that student-athletes would demonstrate greater adherence to the Mediterranean diet but poorer sleep quality, reflecting the higher demands of training and competition. In contrast, we expected no substantial between-group differences in chronotype and body image, as these characteristics are likely inherent to the populations under study.

## 2. Materials and Methods

### 2.1. Participants

Twenty-eight female participants were recruited from the University of Cassino and Lazio Meridionale. In adherence to the Declaration of Helsinki, this cross-sectional study protocol was approved on 8 March 2023 by the Institutional Review Board of the Department of Human Sciences, Society and Health at the University of Cassino and Lazio Meridionale (approval Number 9407) and conducted at the Human Performance Lab (HPL). The total sample (*n* = 28) was divided based on their athletic status. The student-athletes group consisted of volleyball players from the university’s official team (*n* = 12), assessed during their in-season competitive period. The number of participants reflects the usual number of volleyball players on a team. Including an entire team ensured that participants had similar training schedules, competitive experience, and environmental conditions, reducing potential confounding factors that could arise from mixing athletes of different sports or performance levels. Since physical activity and sedentary behaviors are not the opposite of each other, individuals may meet recommended levels of physical activity and still accumulate high sedentary time, which represents an independent risk factor for adverse health outcomes [28]. Moreover, sitting time (>5 h per day) has been used as the primary criterion to identify individuals with sedentary behaviors, independently of their physical activity levels, and the literature highlights its impact on health outcomes such as body image and exercise dependence, and has negative physiological effects [29]. For these reasons, the inclusion of participants in the peer group was independent of their actual levels of physical activity; non-athlete students (*n* = 16) with a high sitting time were included and assessed using the Italian short version (7 items) of the International Physical Activity Questionnaire (IPAQ).

### 2.2. Procedures

Body mass (kg) and height (m) measurements were recorded using a Seca 709 scale equipped with an integrated stadiometer, with precision up to 0.1 kg for weight and 0.1 cm for height (Vogel & Halke, Hamburg, Germany). The body mass index (BMI) was calculated using the formula of weight in kilograms (kg) divided by the square of height in meters (m^2^). All participants were classified as young adults (aged between 18 and 35 years) and had a BMI within the non-clinical range (18.5–29.9 kg/m^2^) [30,31]. The participant’s characteristics are detailed in Table 1.

**Figure 1:**
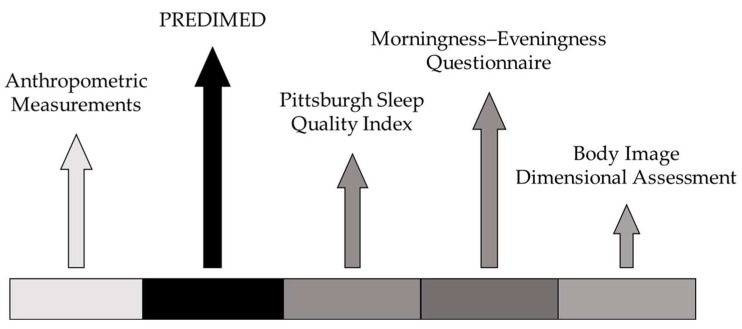
Timeline of experimental procedures. PREDIMED = PREvención con Dieta MEDiterránea.

Data collection was carried out during a single supervised session using standardized procedures and dedicated questionnaires. The procedure’s timeline, lasting around 45 min, is illustrated in Figure 1.

The Italian version of the PREvención con Dieta MEDiterránea (PREDIMED) questionnaire was used to assess adherence to the Mediterranean diet [32]. PREDIMED is a 14-item questionnaire in which an adequate consumption of typical traditional Mediterranean foods and low consumption of foods that are not characteristic of the traditional Mediterranean diet results in one point. The PREDIMED score was calculated as the sum of all the points attributed to the items. Scores ranged from 0 (low adherence) to ≥10 (high adherence).

The Pittsburgh Sleep Quality Index (PSQI) evaluated subjective sleep quality and disturbances over the previous month. It includes 19 questions grouped into seven components: subjective sleep quality, sleep latency, sleep duration, habitual sleep efficiency, sleep disturbance, use of sleeping medications, and daytime dysfunction. Each component is scored on a scale from 0 to 3, with higher scores indicating greater dysfunction. A PSQI total score below 5 indicates good sleep quality, whereas scores ≥ 5 denote poor sleep quality [33].

The Morningness–Eveningness Questionnaire (MEQ) assessed individual preferences for the timing of daily activities. The questionnaire includes both Likert-type and time-based questions. Likert-type items offer four options, with lower scores indicating stronger evening preference. Time-based items are scored based on selected time intervals over a 7 h range, with all responses scored from 1 to 5. The total score is the sum of all item scores and is used to classify chronotype into five categories: definitely morning type (70–86), moderately morning type (59–69), neither type (42–58), moderately evening type (31–41), and definitely evening type (16–30) [34].

Body dissatisfaction was evaluated using the Italian-adapted Body Image Dimensional Assessment (BIDA) [35]. BIDA assesses subjective and emotional dimensions of body image using a neutral silhouette scale. The silhouette-based scale approach was chosen due to its effectiveness in minimizing biases from detailed and/or realistic images, focusing instead on basic body shape perceptions. Participants were asked to select silhouettes that represented their perceived and ideal body shape, the body shape that they believed was most prevalent among their peers, and the body shape they perceived as most attractive to the opposite sex. The scale offered a range of figures depicting different body shapes (extending from 1.8 to 5.2), allowing participants to choose intermediate values. Three indices were calculated:

- Body Dissatisfaction (BD): The difference between perceived and ideal body images.
- Sexual Body Dissatisfaction (SxBD): The difference between perceived body shape and the shape deemed most attractive by the opposite sex.
- Comparative Body Dissatisfaction (CBD): The difference between perceived body image and peers’ typical shape.

Each index is expressed as a percentage that can range from −100% to +100%. Positive values indicate that the participant’s actual rating is higher than desired, compared to what is perceived as sexually attractive, or compared to the average among peers. Conversely, negative values suggest a lower self-assessment. A composite Body Dissatisfaction Index (BDI) was calculated (mean of absolute BD, SxBD, CBD values), with scores above 30% indicating risk for body image disorders.

### 2.3. Statistical Analysis

STATA software version 18 (StataCorp, College Station, TX, USA) was used for statistical analysis. The Shapiro–Wilk test was used to assess the normal distribution of the data. Means and standard deviations were calculated for all variables, while ranges were identified for anthropometric characteristics. One-way ANOVA examined differences between student-athletes and non-athlete students with high sitting times regarding PREDIMED, the PSQI score, the seven component scores of sleep (subjective sleep quality, sleep latency, sleep duration, sleep efficiency, sleep disturbance, use of sleep medication, and daytime dysfunction), MEQ, and BIDA scores. Cohen’s d (*d*) and the 95% confidence interval (95% CI) were used to evaluate the power of all the primary comparisons. In addition to group comparisons using ANOVA, multivariable regression analyses were performed with Heteroscedasticity Consistent (HC3) robust standard errors. Interaction terms were tested to evaluate potential moderation. Results are presented with coefficients (β), 95% CIs, *p*-values, and R^2^. The significance level was set at *p* < 0.05.

## 3. Results

**Figure 2:**
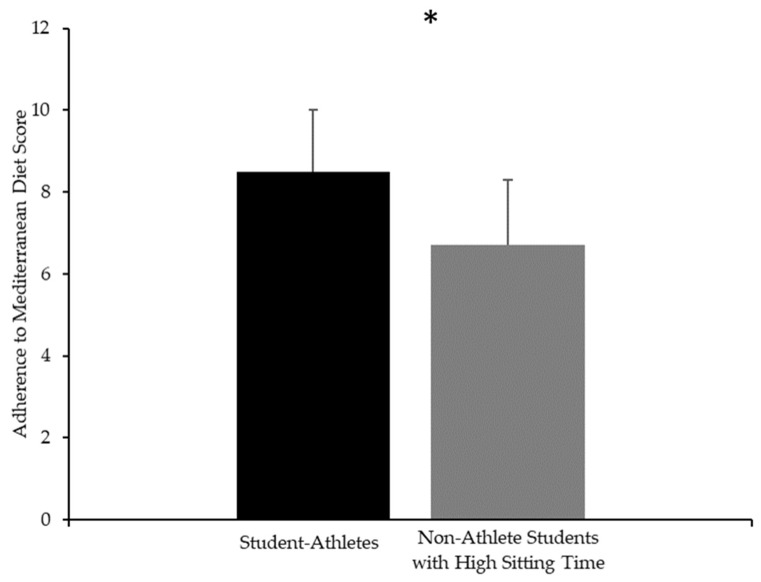
Mean and standard deviation scores of adherence to Mediterranean diet for student-athletes (black) and non-athlete students with high sitting time (gray). * Significant differences between groups (*p* < 0.05).

IPAQ showed that the majority of student-athletes were classified in the HEPA-active category (*n* = 10; 83.3%), while two students (16.7%) were classified as minimally active. In contrast, the non-athlete students with high sitting times showed a more heterogeneous distribution, with seven (43.8%) classified as undertaking HEPA activity, seven (43.8%) as minimally active, and two (12.5%) as inactive. A significant difference between groups emerged for the PREDIMED score (Figure 2), with student-athletes reporting higher (F_(1,26)_ = 8.03, *d* = 1.16 [95%CI: 0.13 to 2.10], *p* < 0.01) adherence to the Mediterranean diet (8.5 ± 1.5 score) compared to non-athlete students with a high sitting time (6.7 ± 1.6 score). The multivariable regression model (Supplementary Table S2) explained 40.8% of the variance (F_(4,23)_ = 2.94, *p* = 0.01, β = 2.24, [95%CI: −3.95 to −0.52]).

**Figure 3:**
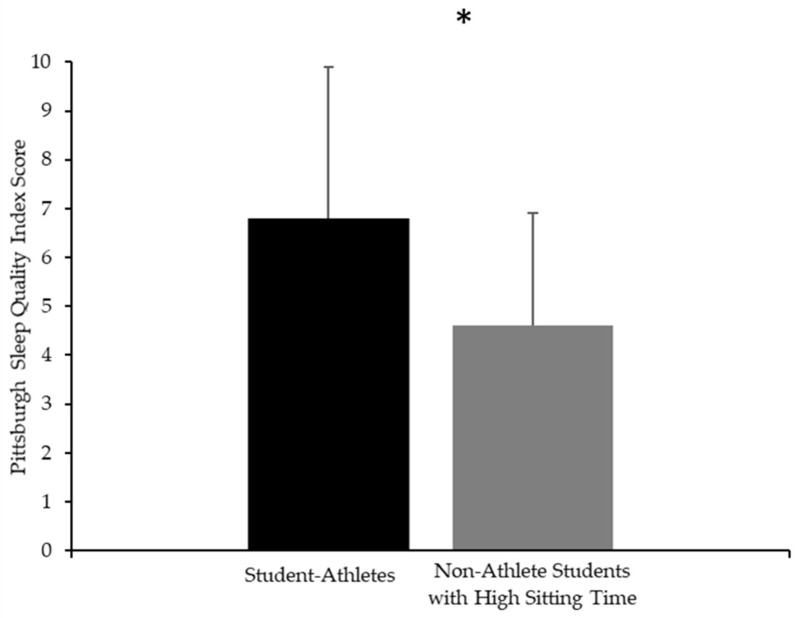
Mean and standard deviation scores of Pittsburgh Sleep Quality Index for student-athletes (black) and non-athlete students with high sitting time (gray). * Significant differences between groups (*p* < 0.05).

Similarly, a significant difference between groups was observed for the PSQI total score (Figure 3), with student-athletes indicating poorer (F_(1,26)_ = 4.69, *d* = 0.82 [95%CI: 0.05 to 1.61], *p* = 0.03) sleep quality (6.8 ± 3.0 score) compared to non-athlete students with a high sitting time (4.6 ± 2.3 score). When applying the clinical cutoff value (PSQI ≥ 5), 75% of the student-athletes (9 out of 12) and 43.8% of the non-athlete students (7 out of 16) were classified as having poor sleep quality. No significant differences were found for any of the seven PSQI components between groups (Supplementary Table S1). Thus, the higher global PSQI score among student-athletes was not driven by a specific component of the PSQI but instead reflected the global effect across domains. The multivariable regression model (Supplementary Table S3) explained 35.5% of the variance (F_(4,23)_ = 3.21, *p* = 0.007, β = −3.46, [95%CI: −5.90 to −1.03]). Moreover, independently of the groups, lower sleep quality was associated with a higher BDI (β = 0.11, [95%CI: 0.00 to 0.21], *p* = 0.04). No significant relationships were found with PREDIMED and MEQ.

**Figure 4:**
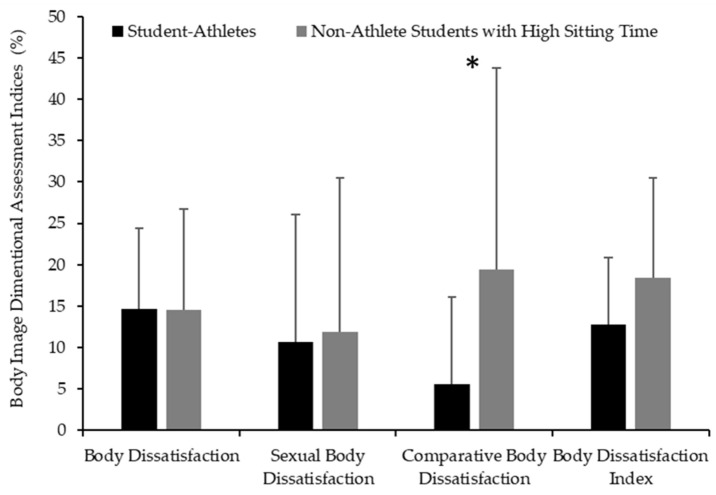
Mean and standard deviation scores of Body Image Dimensional Assessment for student-athletes (black) and non-athlete students with high sitting time (gray). * Significant differences between groups (*p* < 0.05).

A significant difference between groups was also detected for CBD (Figure 4), with student-athletes reporting lower (F_(1,26)_ = 3.31, *d* = −0.67 [95%CI: −1.46 to −0.08], *p* = 0.04) CBD values (5.6 ± 10.5%) than non-athlete students with a high sitting time (19.4 ± 24.5%). The multivariable regression model (Supplementary Table S4) for CBD was not significant overall (F_(4,23)_ = 1.69, *p* = 0.19, R^2^ = 0.25).

**Figure 5:**
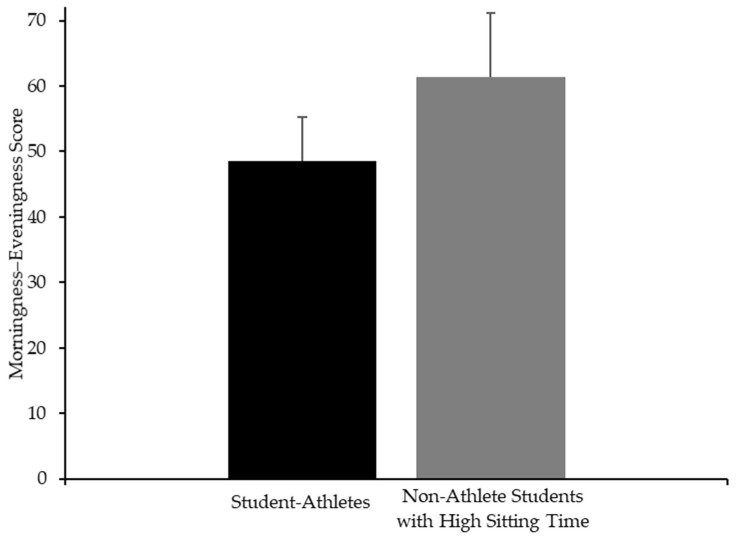
Mean and standard deviation scores of Morningness–Eveningness Questionnaire for student-athletes (black) and non-athlete students with high sitting time (gray).

No significant (*p* < 0.05) differences emerged between groups for the BD (*d* = 0.01 with 95%CI: −0.76 to 0.76), SxBD (*d* = −0.06 [95%CI: −0.81 to −0.03]), or BDI (*d* = −0.51 [95%CI: −1.28 to −0.25]). Regarding chronotype (Figure 5), no significant differences were found between groups (student-athletes: 48.6 ± 6.7 score; non-athlete students with high sitting time: 51.3 ± 9.8 score, *d* = −0.3 [95%CI: −1.06 to 0.44]). Most student-athletes were classified as neither type (91.7%), with only one participant (8.3%) reporting as being a moderately evening type. In contrast, non-athlete students with a high sitting time were more heterogeneous, with 56.3% classified as neither type (9 participants), 18.8% as a moderately morning type (3 participants), and 25% as a moderately evening type (4 participants). The multivariable regression model (Supplementary Table S5) explained 21.1% of the variance (F_(4,23)_ = 1.45, *p* = 0.25). Additionally, PREDIMED significantly predicted chronotypes (β = 2.36, [95%CI: 0.13 to 4.59], *p* = 0.03).

## 4. Discussion

In the present study, we compared female volleyball student-athletes with non-athlete students with a high sitting time to determine whether adherence to the Mediterranean diet, sleep quality, chronotype, and body image differed between the two groups. Our findings highlight that although student-athletes demonstrated significantly higher adherence to the Mediterranean diet, they also reported poorer sleep quality compared to their sedentary peers. This paradox underscores the complexity of lifestyle behaviors among dual-career individuals, emphasizing how concurrent academic and athletic demands might compromise crucial psychophysiological aspects.

Our findings indicate that student-athletes showed higher adherence to the Mediterranean diet than non-athlete students with a high sitting time, which is in line with existing research indicating that athletes, especially within structured team settings, often have greater nutritional awareness and receive specific dietary guidance [21]. Participation in sports likely encourages beneficial health behaviors, including regular meals, appropriate nutrient timing, and selection of whole foods, driven by performance needs. However, the results could also be due to contextual factors, such as food availability and the competitive season. Environmental access (team routines, canteens/meal plans, nutritionist guidance) can facilitate the Mediterranean adherence diet, independent of individual knowledge about its health benefits. Moreover, the athletes were assessed in-season, when diet is often more tightly controlled for performance, although athletes could have a different approach to diet during their off-season. The poorer sleep quality observed among student-athletes raises concerns, since sleep plays a crucial role in physical recovery, cognitive function, and emotional regulation [16,26]. While student-athletes are typically considered healthy, these findings support increasing evidence of vulnerability to sleep disruptions due to factors like training volume schedules, irregular daily routines, pre-competition anxiety, and academic stress [36]. This unexpected pattern suggests that, although athletic participation may confer advantages in other health domains, it can also expose student-athletes to sleep-specific risk factors due not only to the dual-career demands, but also from requirements of specific sports, such as early-morning training, competition-related anxiety, or greater recovery needs, which could further increase the risk of reporting sleep disturbances [37,38]. Moreover, early morning practices and evening competitions may misalign with natural circadian rhythms, particularly in those with an evening chronotype, potentially reducing sleep duration and efficiency [39]. Although previous studies indicate that Mediterranean diet adherence may improve sleep quality [40], our findings suggest that such beneficial effects may be reduced by behavioral and environmental stressors associated with dual-career lifestyles. It is possible that dietary benefits alone cannot fully offset chronic disruptions caused by conflicts between academic/athletic responsibilities and biological sleep preferences. Furthermore, no significant differences were observed between groups regarding chronotype scores, suggesting that external factors, like rigid academic and training schedules, may have a greater impact on sleep than intrinsic biological rhythms, as confirmed by the regression analysis showing that one additional point on the PREDIMED scale is associated with a 2.36 score increase in chronotype, indicating a possible link between healthier nutrition habits and being a morning-type person.

Regarding body image, no significant differences were found between groups for the overall BDI score. However, CBD was significantly higher among non-athlete students with a high sitting time, suggesting they perceive discrepancies between their bodies and peer standards. This difference may reflect the fact that while non-athlete students with a high sitting time might compare themselves to idealized societal or athletic images, student-athletes, being part of an environment focused on physical performance and functionality, could be less influenced by such ideals [41]. The significant difference found in CBD only highlights the importance of considering specific dimensions of body image, rather than referring to a global index when investigating mental health determinants in young adults. The lack of differences in other indices, in particular, BD and the BDI score, could be explained by sex differences in body perception, since females tend to exhibit greater BD than their male counterparts [29,42]. Therefore, future studies should consider comparing sex-related differences in BIDA indices. These alternative interpretations underscore the multifactorial nature of lifestyle behaviors and call for caution when attributing observed differences solely to the dual-career condition, emphasizing the need for a multidimensional understanding of the phenomenon.

Findings from the present study emphasize the importance of integrated health promotion strategies for student-athletes, addressing not only dietary habits and chronotype but also psychological factors, like body dissatisfaction, and sleep hygiene that may involve promoting awareness of circadian rhythms, providing strategies to help balance academic and athletic commitments, and integrating sleep optimization into training objectives. Additionally, institutional policies that acknowledge the challenges of dual careers [43], such as flexible class schedules and sport programs designed to support healthy sleep habits, play a crucial role in creating an environment where student-athletes can effectively balance academic responsibilities and athletic commitments, ultimately promoting both performance and well-being.

## 5. Clinical Implications

The present findings support the development of integrated health strategies for female student-athletes, aiming not only to promote adherence to balanced dietary models such as the Mediterranean diet but also to safeguard sleep hygiene and circadian alignment, crucial for psychophysical recovery and performance. From a clinical and preventive standpoint, the coexistence of healthy eating and poor sleep quality may limit the full benefits of nutritional strategies in athletic populations. Early screening for sleep disturbances, personalized chronotype-based scheduling, and multidisciplinary interventions involving dietitians, coaches, and sleep specialists could help optimize recovery, reduce stress-related symptoms, and prevent performance decrements or long-term health risks. These insights are especially relevant in the context of health promotion among young adult women with high physical and academic demands, underscoring the value of integrative, evidence-based approaches in sports nutrition and preventive medicine.

## 6. Conclusions

This study highlights a paradoxical lifestyle among female student-athletes, emphasizing the importance of integrative health strategies to address both nutrition and sleep, as well as body image concerns, within dual-career contexts. Although exploratory in nature, the present work provides effect-size estimates that may guide future large-scale and longitudinal studies. A stronger focus on multidisciplinary interventions, combining nutritional guidance, sleep hygiene, and psychosocial support, will be essential to promote both well-being and performance in female student-athletes. Our findings also suggest that specific dimensions of body image, rather than global indices, may better capture mental health determinants in young adults. These findings underscore the interconnected nature of behavioral health and highlight the need for comprehensive wellness strategies that align with the realities faced by dual-career individuals. Addressing sleep as a critical area of vulnerability, together with nutrition and psychosocial factors, could enhance overall health, academic success, and athletic performance.

## 7. Limitations and Future Directions

This study presents several limitations, including a relatively small sample size restricted to the female university population, potentially limiting generalizability. Additionally, this cross-sectional study highlights differences between adherence to the Mediterranean diet, sleep quality, chronotype, and body image indices between student-athletes and non-athlete students with a high sitting time, but it cannot determine cause-and-effect relationships. Moreover, the use of self-reported instruments may have introduced potential biases and subjectivity. Future research should explore longitudinal changes in lifestyle factors throughout the academic year, incorporate objective sleep measures (e.g., actigraphy), and examine additional psychological variables such as stress, anxiety, and effective time management strategies. Moreover, we acknowledge that differences in physical activity levels within the non-athlete students could have introduced some heterogeneity, which should be considered when interpreting the findings. Therefore, future studies should aim to separate the roles of sedentary time and physical activity and their effect on health-related outcomes, ideally using larger samples, objective activity monitoring, and stratifying by both sitting time and IPAQ categories. A further limitation concerns the scope of statistical analyses. While group differences are presented with accompanying effect sizes, the sample size did not permit robust multivariate modeling or formal tests of interaction and covariation among variables. Consequently, we could not adjust for potential confounders (e.g., age, BMI, or physical activity level subcategories) or formally explore moderation/mediation effects. These analyses are important for clarifying causal pathways and context-dependent associations and should be prioritized in future studies with larger, multicenter samples using appropriate powered regression-based approaches (e.g., ANCOVA, linear mixed models, and structural equation modeling) to examine interactions, control for covariates, and test for mediation hypotheses.

## Figures and Tables

**Table 1:** Means and standard deviations (SDs) of the participants’ anthropometric characteristics.

	Student-Athletes (*n* = 12)	Non-Athlete Students with High Sitting Time (*n* = 16)	Total (*n* = 28)
	Mean ± SD (Min–Max)	Mean ± SD (Min–Max)	Mean ± SD
Age (years)	21.6 ± 2.4 (19–26)	24 ± 3.2 (20–32)	23 ± 3.1
Body Mass (kg)	64.3 ± 8.1 (52–76)	64 ± 8.2 (52–78)	64.5 ± 8.1
Body Height (cm)	168 ± 7 (159–180)	164 ± 5 (153–177)	166 ± 6
BMI (kg/m^2^)	22.5 ± 2.3 (18.8–25.9)	23.8 ± 2.8 (19.2–28.8)	23.3 ± 2.6

Min: minimum; Max: maximum.

## Data Availability

Data are available in a publicly accessible repository: the original data presented in this study are openly available at https://github.com/ccortis/DataBNR.git.

## Supplementary Materials

The following supporting information can be downloaded at: https://www.mdpi.com/article/10.3390/nu17182946/s1, Table S1: Pittsburgh Sleep Quality Index (PSQI) component scores by group; Table S2: Multivariable regression model for Mediterranean Diet Adherence (PREDIMED) with HC3 robust SEs (N = 28); Table S3: Multivariable regression model for Sleep Quality (PSQI) with HC3 robust SEs (N = 28); Table S4: Multivariable regression model for Comparative Body Dissatisfaction (CBD) with HC3 robust SEs (N = 28); Table S5: Multivariable regression model for Chronotype with HC3 robust SEs (N = 28).
