# Supplementary material for: Eating Right, Sleeping Tight? A Cross-Sectional Study on the Student-Athlete Paradox for Diet and Sleep Behaviors
Source: Nutrients. 2025 Sep 12;17(18):2946. doi: 10.3390/nu17182946 (PMC12472328; doi:10.3390/nu17182946)
Supplement: Supplementary file 1 [file nutrients-17-02946-s001.zip › nutrients-3833935-supplementary.pdf]

Supplementary Table S1. Pittsburgh Sleep Quality Index (PSQI) component scores by group

PSQI Component	Student-Athlete (n=12)	Non-Athlete Students (n=16)
Subjective sleep quality	1.33 ± 0.5	0.94 ± 0.8
Sleep latency	1.67 ± 1	1.19 ± 1.1
Sleep duration	0.58 ± 0.7	0.25 ± 0.6
Sleep efficiency	0.67 ± 1	0.19 ± 0.4
Sleep disturbance	1.33 ± 0.6	1.13 ± 0.3
Use of sleep medication	0.33 ± 0.8	0 ± 0
Daytime dysfunction	0.92 ± 0.8	0.94 ± 0.7

Supplementary Table S2. Multivariable regression model for Mediterranean Diet Adherence (PREDIMED) with HC3 robust SEs (N = 28)

Predictor (IV)	β	SE	95% CI	p-value	R^2
Group (sedentary vs athlete)	–2.24	0.83	–3.95 to –0.52	0.01	0.41
Sleep quality (PSQI)	–0.13	0.16	–0.46 to 0.20	0.4	
Chronotype	0.08	0.04	–0.00 to 0.16	0.05	
Body dissatisfaction (BDI)	–0.00	0.04	–0.08 to 0.08	0.9	

Supplementary Table S3. Multivariable regression model for Sleep Quality (PSQI) with HC3 robust SEs (N = 28)

Predictor (IV)	β	SE	95% CI	p-value	R^2
Group (sedentary vs athlete)	–3.46	1.18	–5.90 to –1.03	0.007	0.36
Diet adherence (PREDIMED)	–0.35	0.36	–1.09 to 0.40	0.3	
Chronotype	0.02	0.05	–0.09 to 0.12	0.7	
Body dissatisfaction (BDI)	0.11	0.05	0.00 to 0.21	0.04	

Supplementary Table S4. Multivariable regression model for Comparative Body Dissatisfaction (CBD) with HC3 robust SEs (N = 28)

Predictor (IV)	β	SE	95% CI	p-value	R^2
Group (sedentary vs athlete)	9.64	7.93	–6.77 to 26.05	0.2	0.25
Diet adherence (PREDIMED)	–3.05	3.06	–9.38 to 3.28	0.3	
Sleep quality (PSQI)	1.43	1.43	–1.53 to 4.40	0.3	
Chronotype	0.74	0.65	–0.60 to 2.09	0.2	

Supplementary Table S5. Multivariable regression model for Chronotype with HC3 robust SEs (N = 28)

Predictor (IV)	β	SE	95% CI	p-value	R^2
Group (sedentary vs athlete)	6.78	4.64	–2.82 to 16.37	0.1	0.21
Diet adherence (PREDIMED)	2.36	1.08	0.13 to 4.59	0.03	
Sleep quality (PSQI)	0.17	0.61	–1.10 to 1.44	0.7	
Body dissatisfaction (BDI)	0.07	0.20	–0.35 to 0.48	0.7	
